# Analysis of SEC24D Gene in Breast Cancer Based on UALCAN Database

**DOI:** 10.1515/biol-2019-0080

**Published:** 2019-12-31

**Authors:** Zhi Liu, Jing Zhou, Zhibao Wang, Zhiqiang Zhou

**Affiliations:** 1 Department of Radiology, Affiliated Hospital of North China University of Science and Technology, Tangshan 063000, P.R. China; 2 Department of Endocrinology, Tangshan Hospital of Traditional Chinese Medicine, Tangshan 063000, P.R. China; 3 Department of Radiology, The No.2 Hospital of Baoding, Baoding 071051, P.R. China

**Keywords:** Breast cancer, SEC24D, UALCAN, Prognosis

## Abstract

**Objective:**

To analyze the expression and its clinical significance of the SEC24D gene in breast cancer.

**Methods:**

The dataset of breast cancer were searched in the UALCAN database, and the data obtained were mined and combined with literature analysis.

**Results:**

The mRNA expression of the SEC24D gene in breast cancer tissues was significantly higher than that of breast normal tissues from the UALCAN database (*P* < 0.05). The promoter methylation levels of the SEC24D gene in breast cancer tissues were lower than that of breast normal tissues (*P* < 0.05). Survival analysis showed that the relapse-free survival of breast cancer patients with a higher expression of SEC24D gene was significantly worse than those patients with a lower expression of SEC24D (*P* < 0.05).

**Conclusion:**

The SEC24D gene has a high expression in breast cancer tissues and its expression level was related to the prognosis of breast cancer patients.

## 1 Introduction

In recent years, the global incidence of breast cancer has been on the rise and has become a major public health problem in society [1, 2]. Breast cancer is one of the most common malignant tumors in women. Its incidence rate ranks first among women’s common tumors, and mortality ranks second [3], second only to lung cancer, which seriously threatens women’s health. Therefore, it is necessary to continue to study the molecular mechanism of breast cancer development and to find therapeutic targets and prognostic molecular markers.

Genomics data from the Cancer Genome Atlas (TCGA) project led to comprehensive molecular features of various cancer types. TCGA’s large sample data provides an excellent opportunity to solve problems related to heterogeneity. Various computing tools are developed by TCGA aid staff to perform specific data analysis. UALCAN is an easy to use, interactive web-portal to perform to in-depth analyses of TCGA gene expression data.

The SEC24D gene is a member of the SEC24 gene subfamily [4]. The protein encoded by this gene is a component of COPII (Coat Protein Complex II), and COPII can mediate endoplasmic reticulum synthesis of proteins for transport to the Golgi apparatus [5]. Experimental studies found that when knocking out the SEC24 gene of zebrafish, COPII could not be formed, protein transport from the endoplasmic reticulum to the Golgi apparatus was blocked, and the naive protein was accumulated in the endoplasmic reticulum, resulting in abnormal cartilage morphology and craniofacial malformation of zebrafish [6]. Clinically, it has also been found that mutations in the SEC24D gene in the human population can cause osteogenesis imperfecta [7]. Intracellular protein transport is also an important link affecting tumor cell viability and proliferation [8], and SEC24D gene is an important factor affecting protein transport, suggesting that the SEC24D gene plays an active role in tumor cells. However, the author has not seen such research reports yet. This study then collected and analyzed common data sets in the UALCAN database to understand the expression and clinical significance of the SEC24D gene in breast cancer.

## 2 Materials and Methods

### 2.1 The UALCAN database

UALCAN database is a portal for facilitating tumor gene expression and survival analyses [9]. In this database, you can set the conditions for filtering and data mining. The screening conditions set in this study are: “Gene: SEC24D”; “Analysis Type: Breast Cancer vs. Normal Analysis”; “Cancer Type: Breast invasive carcinoma”; “Data Type: TCGA dataset”.

### 2.2 The Kaplan-Meier Plotter

Online survival analysis was performed using the breast cancer dataset of the Kaplan-Meier Plotter (KM Plotter) [10]. The screening conditions are as follows: “Cancer: Breast Cancer”; “Gene symbol: SEC24D”; “Affy id: 202375_ at”; “Survival: Relapse free survival”.

### 2.3 Statistical processing

The difference in SEC24D gene expression between breast cancer and normal tissue was analyzed by t-test. The relationship between SEC24D gene expression and prognosis was analyzed by Kaplan-Meier model. The survival rate between the two groups was compared by Log-Rank test. The test level was α=0.05. The data used were analyzed by online statistical analysis. P<0.05 was considered statistically significant.

## 3 Results

### 3.1 Pan-cancer view of SEC24D expression level

**Figure 1:**
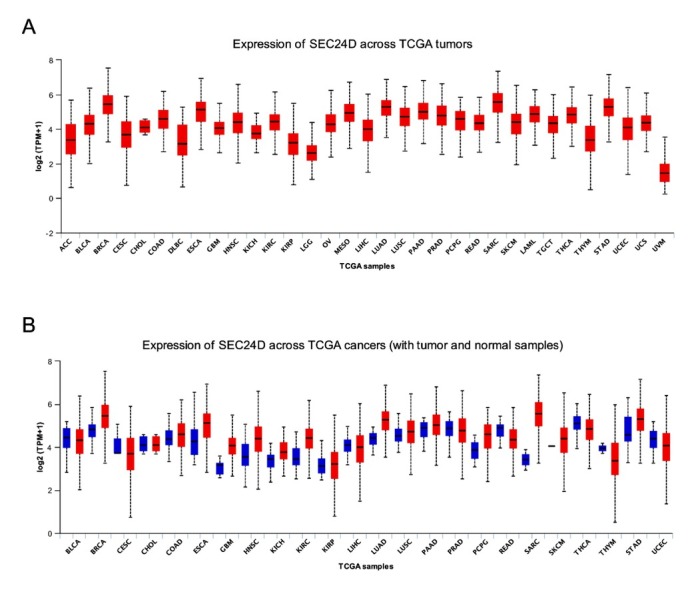
Pan-cancer view of SEC24D expression level

Expression of SEC24D across TCGA tumors was showed in **Figure 1A**. The result indicated that the expression level of SEC24D was the highest in all TCGA tumors. The expression difference of SEC24D with tumor and normal samples was shown in **Figure 1B**. The result indicated that the expression level of SEC24D in tumor tissue was higher than its matched normal tissue in most of the tumors.

### 3.2 Expression of SEC24D in breast cancer

**Figure 2:**
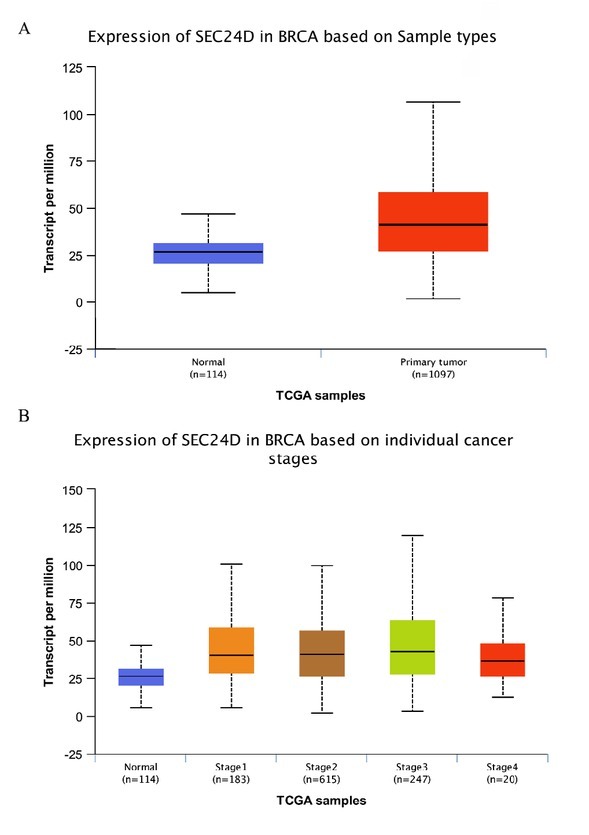
Expression of SEC24D in breast cancer

Statistical analysis in the UALCAN database revealed that SEC24D was highly expressed in breast cancer compared with the normal group, and the difference was statistically significant (P<0.05), as shown in **Figure 2A**. Further analysis found that the expression of SEC24D was higher in breast cancer based on individual cancer stages than normal tissues, and the difference was statistically significant (P<0.05), as shown in **Figure 2B**.

### 3.3 Promoter methylation level of SEC24D in breast cancer

**Figure 3:**
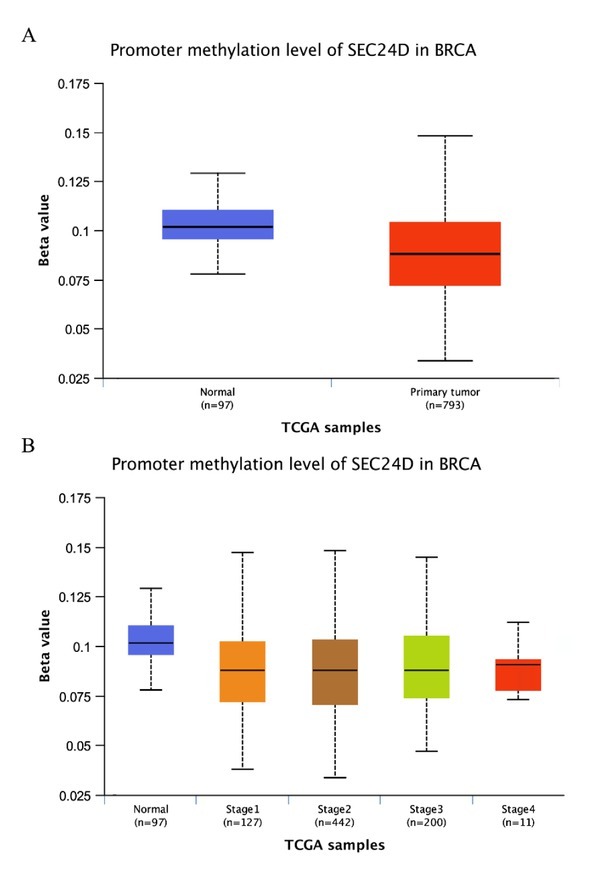
Promoter methylation level of SEC24D in breast cancer

The level of methylation in the promoter region is closely related to the development of tumors. Therefore, we analyzed the methylation level of the SEC24D promoter region in breast cancer tissues. We found that the promoter methylation level of SEC24D in breast cancer was more obviously reduced than normal tissues, as shown in **Figure 3A**. Further analysis found that the promoter methylation level of SEC24D was lower in breast cancer based on individual cancer stages than normal tissues (**Figure 3B**). The above results indicate that the expression of SEC24D may be related to the degree of methylation in the promoter region.

### 3.4 Relationship between SEC24D expression level and prognosis of patients with breast cancer

**Figure 4:**
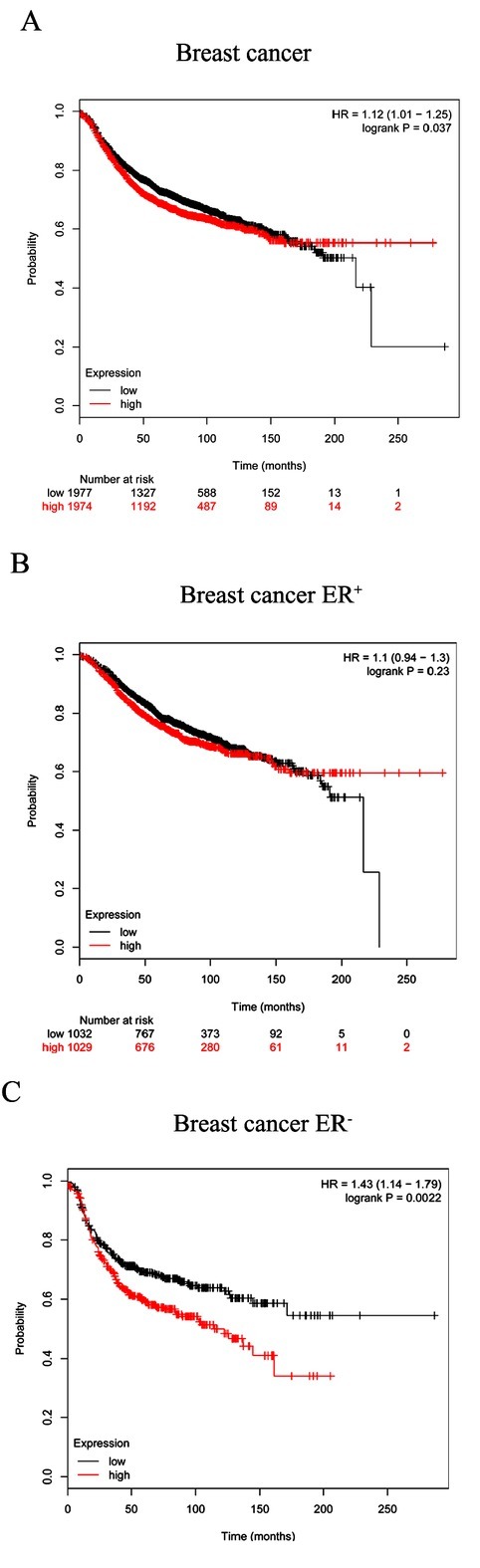
Relationship between SEC24D expression level and prognosis of patients with breast cancer

To further clarify the relationship between SEC24D expression level and the prognosis of patients with breast cancer, the KM Plotter database analysis showed a survival curve of 2519 patients with high SEC24D expression and low expression group, and found that the SEC24D expression level has a significant impact on relapse-free survival of patients. Compared with 1977 patients with low-expression, 1974 patients with high SEC24D expression had significantly lower RFS (HR=1.12, logrank P=0.037, **Figure 4A**). This indicates that SEC24D expression is a risk factor for prognosis in patients with breast cancer. Further subgroup analysis showed that in 801 patients with ER^-^ breast cancer, high RPA3 expression levels had a significant effect on RFS (HR=143, logrank P=0.0022, **Figure 4C**), whereas in 2061 patients with ER^+^ breast cancer, its high expression level had no significant effect on RFS (HR=1.1, logrank P=0.23, Figure 4B).

## 4 Discussion

Studies have shown that the new synthesized secreted proteins in the endoplasmic reticulum must be transported to the Golgi apparatus by means of small vesicles composed of COPII for processing and modification, and then transported to specific parts of the cells or secreted outside the cell, thus COPII plays a vital role in intracellular protein transport [11]. The inner layer of COPII is a heterodimer composed of SEC23A and SEC24D proteins. The mutation or deletion of the SEC24D gene will directly affect the formation of COPII, hindering the transport of intracellular proteins [12]. The synthesis and transportation of proteins in tumor cells is particularly strong, and it is a feasible method to seek an anti-cancer pathway from the perspective of inhibiting the synthesis and transportation of tumor cell proteins [13]. However, current research on the mechanism of action against tumors still focuses on inhibiting proliferation, inducing apoptosis [14], influencing cell cycle [15], inhibiting invasion and metastasis, inhibiting signal transduction [16], inhibiting vascular mimicry and angiogenesis [17], and immunity. This route of transportation has not yet received enough attention. This study is based on this idea, using data mining methods to analyze the expression of the SEC24D gene in breast cancer in a large sample of many researchers to avoid biased conclusions due to differences in research methods, sample size and population.

As we all know, the TCGA database is the world’s largest and most comprehensive gene chip database [18]. So far, TCGA researchers have analyzed large cohorts of over 30 human tumors through large-scale genome sequencing and integrated multi-dimensional analyses [19]. UALCAN is a data extraction platform based on the TCGA database. UALCAN provides in-depth analysis of TCGA gene expression profile data. UALCAN accesses TCGA’s Level 3 RNA sequencing and clinical data for 31 cancer types [20], and can perform the following analysis:

differential expression of query genes between tumors and normal tissues, based on tumor stage, grade, patient ethnicity, and various other clinicopathological features; Differential expression of query genes among tumor subgroups; assessment of the effect of gene expression levels and clinicopathological features on patient survival; identification of up-regulated and down-regulated genes in individual cancer types. The portal serves as a platform to validate the expression of target genes and identify tumor subgroup-specific candidate biomarkers.

In this study, we deeply explored the gene expression profile data of breast cancer and found that the expression of the SEC24D gene in breast cancer tissues was significantly higher than that in adjacent tissues. The survival data by Kaplan-Meier method showed that the disease-free survival of breast cancer patients with a high expression of the SEC24D gene was significantly worse than that of low-expression patients, that is, the prognosis of patients with low expression was better. Combined with the above description of the function of the SEC24D protein, it can be preliminarily speculated that the SEC24D gene can promote the development of breast cancer, and its mechanism may be related to the formation of intracellular protein transfer, which is conducive to the normal transport and secretion of proteins in cancer cells. This mechanism is different from previous anti-cancer research perspectives, so it is expected to become a new target for breast cancer treatment and a new indicator of prognosis.

## References

[1] Swarna LB, Mavuluri J, Gnana VS, Lamjed M, Saleh A, Abdel HH (2019). Andrographolide as a therapeutic agent against breast and ovarian cancers. Open Life Sci.

[2] Ravdin P, Cronin K, Howlader N, Berg C, Chlebowski R, Feuer E (2007). The Decrease in Breast-Cancer Incidence in 2003 in the United States. N Engl J Med.

[3] Siegel R, Desantis C, Virgo K, Stein K, Mariotto A, Smith T (2014). Cancer treatment and survivorship statistics, 2012. Ca Cancer J Clin.

[4] (2015). SEC23B Sec23 homolog B (S. cerevisiae).

[5] Kajiwara K, Ikeda A, Aguileraromero A, Castillon GA, Kagiwada S, Hanada K (2014). Osh proteins regulate COPII-mediated vesicular transport of ceramide from the endoplasmic reticulum in budding yeast. Journal of Cell Science.

[6] Townley AK, Feng Y, Schmidt K, Carter DA, Porter R, Verkade P (2008). Efficient coupling of Sec23-Sec24 to Sec13-Sec31 drives COPII-dependent collagen secretion and is essential for normal craniofacial development. Journal of Cell Science.

[7] Jiang X, Liu L, Zhang Q, Jiang Y, Huang J, Zhou H (2016). Laparoscopic versus open hepatectomy for hepatocellular carcinoma: long-term outcomes. Journal of BU ON: official journal of the Balkan Union of Oncology.

[8] Zhan Y, Sun C, Cao Z, Bao N, Xing J, Lu C (2012). Release of Intracellular Proteins by Electroporation with Preserved Cell Viability. Analytical Chemistry.

[9] Chandrashekar DS, Bashel B, Balasubramanya SAH, Creighton CJ, Ponce-Rodriguez I, Chakravarthi B (2017). UALCAN: A Portal for Facilitating Tumor Subgroup Gene Expression and Survival Analyses. Neoplasia (New York, NY).

[10] Györffy B, Lanczky A, Eklund AC, Denkert C, Budczies J, Li Q (2010). An online survival analysis tool to rapidly assess the effect of 22,277 genes on breast cancer prognosis using microarray data of 1,809 patients. Breast Cancer Res Treat.

[11] Miller EA, Schekman R (2013). COPII - a flexible vesicle formation system. Current opinion in cell biology.

[12] Tomoishi S, Fukushima S, Shinohara K, Katada T, Saito K (2017). CREB3L2-mediated expression of Sec23A/Sec24D is involved in hepatic stellate cell activation through ER-Golgi transport. Sci Rep.

[13] Cunha EFFD, Ramalho TC, Mancini DT, Fonseca EMB, Oliveira AA (2010). New approaches to the development of anti-protozoan drug candidates: a review of patents. Journal of the Brazilian Chemical Society.

[14] Shi L, Fei X, Wang Z (2015). Demethoxycurcumin was prior to temozolomide on inhibiting proliferation and induced apoptosis of glioblastoma stem cells. Tumour Biology the Journal of the International Society for Oncodevelopmental Biology & Medicine.

[15] Hong BS, Cho JH, Kim H, Choi EJ, Rho S, Kim J (2009). Colorectal cancer cell-derived microvesicles are enriched in cell cycle-related mRNAs that promote proliferation of endothelial cells. Bmc Genomics.

[16] Bianco R, Melisi D, Ciardiello F, Tortora G (2006). Key cancer cell signal transduction pathways as therapeutic targets. European Journal of Cancer.

[17] Li W, Miao S, Miao M, Li R, Cao X, Zhang K (2016). Hedgehog Signaling Activation in Hepatic Stellate Cells Promotes Angiogenesis and Vascular Mimicry in Hepatocellular Carcinoma. Cancer Investigation.

[18] Liu Y, Peng Q (2012). Integrative analysis of methylation and gene expression data in TCGA. IEEE International Workshop on Genomic Signal Processing & Statistics.

[19] Katarzyna T, Patrycja C, Maciej W (2015). The Cancer Genome Atlas (TCGA): an immeasurable source of knowledge. Contemporary Oncology.

[20] Rahman M, Jackson LK, Johnson WE, Li DY, Bild AH, Piccolo SR (2015). Alternative preprocessing of RNA-Sequencing data in The Cancer Genome Atlas leads to improved analysis results. Bioinformatics.

